# Inflammatory Bowel Disease and Its Unique Impact on Body Image: A Qualitative Exploration

**DOI:** 10.1002/jclp.70184

**Published:** 2026-07-15

**Authors:** Mia L. Pellizzer, Réme Mountifield, Ruth Prosser, Ivanka Prichard

**Affiliations:** ^1^ Flinders University Institute of Mental Health and Wellbeing Adelaide SA Australia; ^2^ Blackbird Initiative Flinders University Adelaide SA Australia; ^3^ Department of Gastroenterology Flinders Medical Centre Adelaide SA Australia; ^4^ Caring Futures Institute, Embrace Impact Lab Flinders University Adelaide SA Australia

**Keywords:** body image, body image dissatisfaction, functionality appreciation, inflammatory bowel disease, qualitative

## Abstract

**Objective:**

There is increasing interest in the relationship between inflammatory bowel disease (IBD) and body image. Quantitative research demonstrates a high level of body image dissatisfaction among individuals with IBD that is related to poorer quality of life and mental health disorders including depression, anxiety, and eating disorders. However, there remains a gap in our understanding of how patients with IBD qualitatively experience their bodies. The aim of this study was to qualitatively explore the impact IBD has on body image and understand the nuances specific to this population.

**Method:**

Data from co‐design workshop interviews with 10 people with IBD (8 women and 2 men, mean age 35) were analysed using reflexive thematic analysis.

**Results:**

Four main themes were identified: Not Mainstream Body Image; Functionality at the Core of Body Evaluation and Quality of Life; Eating, Symptom Control, and Body Image; and Unpredictability, Invisibility, and Social Appraisals.

**Conclusions:**

Themes captured the unique, pervasive impact IBD has on body image, quality of life, eating behaviour, and mental health. Results highlight the need to target body image concerns and mental wellbeing for people with IBD.

## 1 Introduction

Inflammatory bowel disease (IBD), including Crohn's disease (CD) and ulcerative colitis (UC), encompasses chronic conditions that cause inflammation in the bowel (Baumgart and Carding 2007). Symptoms vary and can include abdominal pain, diarrhoea, rectal bleeding, fatigue, and weight loss (Li et al. 2024). Patients may also experience extraintestinal manifestations (EIMs; symptoms that occur outside of the gastrointestinal tract), commonly affecting the skin, joints, and eyes (Li et al. 2024) with physical, psychological, and appearance impacts. Treatments are focused on inducing and maintaining remissions and typically involve medication (e.g. steroids), surgery, or both, in addition to nutrition and psychotherapy intervention (Li et al. 2024). Treatments have appearance implications, such as weight gain from steroid medication and surgical treatments that can involve the formation of either temporary or permanent stomas (an opening in the abdomen connected to the bowel to remove faeces). Worldwide, IBD is estimated to impact 0.3% of people in Western countries (Kaplan 2015; Ng et al. 2017). In Australia, 653 patients per 100,000 had a diagnosis of IBD when reviewing general practice records (Busingye et al. 2021). Economic evaluations estimate an annual expenditure in excess of $100 million in total hospital costs and $380 million in productivity loss in 2012 (PricewaterhouseCoopers Australia 2013), and $2.7 billion in costs related to IBD management in 2005 (Access Economics 2007). Quality of life is significantly impaired for patients with IBD, including disruptions to work performance and less engagement in leisure pursuits (Ghosh and Mitchell 2007).

Patients with IBD often present with mental health challenges, including mood and anxiety disorders and sleep difficulties across a range of prospective, cross‐sectional, and longitudinal studies (see Balestrieri et al. 2023 for a narrative review). A related, under‐addressed area is the relationship between IBD and body image. Body image is a multifaceted, umbrella construct that encompasses thoughts, feelings, and perceptions about the body and how we act in relation to these thoughts (Cash and Smolak 2011). Body image comprises the related but distinct constructs of positive body image and negative body image (Tylka and Wood‐Barcalow 2015). While positive body image encompasses concepts such as body appreciation, flexibility, and functionality, negative body image is a more maladaptive relationship with one's body, commonly conceptualised as body dissatisfaction (not liking the appearance one's body) (Thompson and Schaefer 2019; Tylka and Wood‐Barcalow 2015). Among patients with IBD, a systematic review of 57, predominantly cross‐sectional studies (of which 31 reported prevalence) found prevalence rates for body dissatisfaction vary from 21% to 81% (Beese et al. 2019). This systematic review also found factors including female gender, age (younger i.e. adolescents), fatigue, disease activity, and steroid use to be associated with greater body dissatisfaction and poorer quality of life (Beese et al. 2019). Given the impacts on appearance of many IBD symptoms (such as weight loss, bloating, and skin changes from EIMs) and treatment side effects (scars from surgery, stoma, weight gain, acne), the connection with body dissatisfaction is unsurprising.

The current literature on body image and IBD has exclusively focused on body dissatisfaction, presenting a gap in how positive body image constructs may be represented in this population. For instance, body functionality appreciation (Alleva and Tylka 2021) is the appreciation of and gratitude towards everything the body is capable of doing across a variety of domains. Body functionality appreciation is an area of increasing interest in positive body image (Alleva and Tylka 2021) and chronic illness populations (e.g., cancer; Brkic et al. 2024). As such, exploring how people with IBD can appreciate their body's functionality in other areas may be of relevance to people with IBD. Body neutrality (Pellizzer and Wade 2023), a concept popular in mainstream media and social media, may also be of relevance. Related to and overlapping with positive body image, body neutrality encourages a neutral and flexible approach to one's body, respect and care of the body, and acknowledging that self‐worth is comprised of intrinsic and extrinsic pursuits, rather than focused solely on one's appearance (Pellizzer and Wade 2023). Body neutrality may be particularly beneficial to explore given it de‐emphasises appearance, which is a particular source of dissatisfaction for people with IBD (Beese et al. 2019).

In the general population using cross‐sectional and longitudinal designs, body dissatisfaction is linked to a host of negative outcomes such as lower exercise engagement, poorer quality of life, low self‐esteem, and increased depression, smoking, and disordered eating (Kilpela et al. 2015; Mellor et al. 2010; Mond et al. 2013). Body dissatisfaction is also an established risk factor for the development of disordered eating, demonstrated in meta‐analytic research (e.g., see Stice 2002). Among patients with IBD, Beese et al.'s (2019) systematic review found body dissatisfaction is also linked to lower quality of life Alarmingly, several cross‐sectional studies have found that patients with IBD often meet cutoff scores on eating disorder screening measures, ranging from 13% (Wabich et al. 2020), 20% (Satherley et al. 2016), and 29% (CD patients, binge eating specific measure; Wardle et al. 2018). This exceeds the global average lifetime prevalence for eating disorders in the general population, which is estimated to be 2.58% for women and 0.74% for men (Qian et al. 2022). A systematic review of IBD and eating disorders, found the combination of CD and anorexia nervosa to be most frequently reported comorbidity (Ilzarbe et al. 2017). A large cross‐sectional study with 1589 patients with IBD found people with IBD are particularly at risk for developing an eating disorder if they are female, dissatisfied with their weight and/or trying to lose weight, have an eating disorder history, and have had a higher number of IBD surgeries (Stoleru et al. 2022). Clinically, our patients have described IBD‐specific interactions with body image, for instance body image impacting marriages, dating, and sex lives. This is an important, underexplored area that requires academic exploration. Taken together, people with IBD experience high rates of body image dissatisfaction, disordered eating and eating disorders, further impairing their quality of life and elevating psychological distress.

To date, a small body of research has qualitatively examined the experience of IBD and psychological health and well‐being. Fourie et al. (2018) conducted a thematic synthesis of 23 qualitative studies focused on living with IBD. Fourie et al. (2018) synthesised five themes, including “living with exhaustion”, “living in a flawed body”, “living in secrecy”, “living with restriction”, and “living in fear”, capturing constant fatigue, poor body image, having an invisible illness with minimal public awareness, managing dietary constraints and quality of life impairments, and overwhelming unpredictability and fear of the unknown (Fourie et al. 2018). “Living in a flawed body” specifically captured frustrations with body functionality, including toileting issues and specific body image concerns related to IBD treatments, such as having puffy cheeks (Fourie et al. 2018).

More recently, Erdmann et al. (2024) qualitatively explored how patients with IBD experience their body, self, and life plans in the larger context of precision medicine and living the “good life”. They identified five themes that tapped into the impact IBD has on quality of life, including: “1) life options and plans, 2) other people's responses, 3) strategies to deal with others' responses, 4) the perception of the body and self, and 5) perception of life as good despite suffering” (Erdmann et al. 2024). One of their identified themes (perception of the body and self) specifically addressed changing body image related to IBD (such as weight changes), feeling unfeminine or unmasculine as a result of changed body appearance, feeling frustrated and angry with one's body, and mental health challenges such as viewing oneself as weak or less worthy than others (Erdmann et al. 2024).

To our knowledge, only one published qualitative study on a sample of 16 patients with IBD from China has explicitly explored the relationship between IBD and body image (Ruan et al. 2020). Six key themes were extracted: “being a constrained person”, “being a flawed person”, “being a disliked person”, “being an alienated person”, “being a reconciled person” and “being a blessed person” (Ruan et al. 2020). These themes encompassed difficulties with body functionality and unpredictability of IBD symptoms and the impact on quality of life and mental health; specific body image dissatisfaction in areas such as weight loss, hair loss, and having a “moon face”; feeling disgust in self and from others due to having IBD such as feeling “dirty” and “useless”; and having a misunderstood illness (Ruan et al. 2020). However, themes also captured acceptance of IBD and positive experiences, such as paying more attention to one's health and feeling stronger as a person due to the challenging experience of IBD (Ruan et al. 2020). Two participants explicitly spoke about liking IBD‐related weight loss and feeling it made them more attractive (Ruan et al. 2020). While framed as a positive experience, this is problematic and may indicate screening for an eating disorder is warranted. Given differences in medical systems as well as cultural ideals of beauty and media influences (Jackson et al. 2016) it is important for research to be conducted in other cultures, such as Western societies like Australia.

In summary, the connection between IBD and body image has been documented in quantitative studies however minimal qualitative insights are available. Given the high rates of IBD in Western societies, it is important to further explore the body image experiences of people with IBD in order to be able to develop tailored interventions to improve body image and wellbeing. Therefore, the aim of the present study was to understand how IBD impacts body image among people with IBD in Australia.

## 2 Method

### 2.1 Participants

A total of 10 people contributed their experiences of body image in the context of having IBD. Their data was collected from a larger study that aimed to co‐design a new intervention targeting body image and wellbeing for people with IBD. For further information about co‐design methodology and results of the co‐design process, see Pellizzer et al. (2025). Participants attended online (*n* = 5) or in person (*n* = 5). Most participants were female, White, heterosexual, and had CD. Full demographic and IBD information are provided in Table 1.

**Table 1:** Demographic and IBD Information.

	*N*
Gender	10
Female	8
Male	2
Cultural Identity	
White	8
Asian	2
Sexuality	
Heterosexual	9
Queer	1
Age (years)	
M (SD)	35.40 (7.86) years Range 19–43 years
Highest Level of Education Completed	
Secondary	3
Vocational or similar	2
University – Undergraduate	4
University – Postgraduate	1
Diagnosis	
Crohn's Disease	7
Ulcerative Colitis	3
Age of Diagnosis	
M (SD)	22.5 (9.02) years Range = 10–37 years
Receiving Current Medical Treatment	
Yes	9
No	1
Previous Bowel Surgery	
Yes	7
No	3
Part of Bowel Affected	
Large bowel	5
Small bowel	1
Both large and small bowel	1
Large and small bowel and stomach	1
Perianal disease	1
No answer provided	1

### 2.2 Procedure

Following approval by the Southern Adelaide Clinical Human Research Ethics Committee (project number 2023/HRE00016), participants were recruited via the Flinders Medical Centre IBD service in Adelaide, Australia. Participants were eligible if they had a diagnosis of IBD, were over the age of 18, proficient in English, and obtained a score of 10 or more on the Body Image Scale (BIS; McDermott et al. 2013). Participants first completed an online questionnaire containing the participant information sheet, consent form, demographic questions (age, gender, cultural identity, sexuality, education), IBD diagnosis and treatment questions (IBD type, age of diagnosis, current treatment, part of bowel impacted), BIS measure, and a question regarding their availability to attend either in person groups or online (via Microsoft Teams) on the specified dates in May and June 2023. Participants completed three workshops of 3 h duration and were reimbursed $40AUD per hour to honour their lived experience expertise. Data from the first two workshops, where there was a discussion of body image, were analyses for the current study. Workshop 1 included an in‐depth discussion of how IBD impacts body image and quality of life and wellbeing more broadly. Prior to this workshop, participants received a handout to define key body image terms and also a lay summary of a literature review examining positive body interventions for the general population (Guest et al. 2019). There was no question guide. It was a largely unstructured, organic conversation that followed the directions of participants, starting with the broad question “how do you think having IBD impacts your body image?”. Workshop 2 reviewed the key points from this discussion, and participants were invited to share any additional content on this area if they wished. Workshop 3, which was focused on reviewing and collating feedback on a prototype version of the intervention, was not subject to qualitative data analysis.

The first author MP, a clinical psychologist with expertise in body image, eating disorder, and co‐design, ran the workshops and had no prior relationships with any of the participants. To facilitate sharing, participants in each group collaboratively created group norms (such as keeping information confidential, being non‐judgemental) and participants were told they could contribute as they wished (i.e. no pressure to answer if not feeling comfortable) and could take breaks and leave the room/Teams meeting as they liked. All participants contributed and reported feeling comfortable.

All qualitative data were collected via Microsoft Teams using the inbuilt transcription function. For both groups (in person or online), Microsoft Teams was set up to record only the relevant conversations in workshops 1 and 2 (described above). A research assistant reviewed each transcript and made adjustments as required. The participant information sheet and consent form specified that workshops would be recorded, and data used in a de‐identified way in resulting publications. Prior to each recording, an overview of the procedure was provided, and consent was confirmed verbally by all participants.

### 2.3 Measures

#### 2.3.1 Body Image Scale (BIS)

The BIS was completed as a screening tool to establish eligibility for participation. This version by McDermott et al. (2013) is specific to people with IBD and demonstrates good reliability and validity. A total score is calculated by summing items, such that higher scores indicate greater body image dissatisfaction. In this study, internal consistency was *α* = 0.75, acceptable but lower than what was reported by McDermott et al. (2013; α = 0.93).

### 2.4 Data Analysis

#### 2.4.1 Reflexive Thematic Analysis

Discussions about the impact of IBD on body image from workshop 1 and the beginning of workshop 2 (from both the online and in person groups) were analysed using reflexive thematic analysis, as described by Braun and Clarke (2022). A critical realist epistemological framework was applied; acknowledging data is situated and interpreted within the influences of language and culture (Braun and Clarke 2022). Authors MP and IP independently completed the first three phases of dataset familiarisation, coding, and generating initial themes. Coding followed an inductive, semantic approach which allows participants explicit meanings to be captured rather than following a pre‐existing researcher approach or theory. The authors then collaborated for the three later phases to develop and review themes, refine themes, and lastly define and name themes. There were minimal discrepancies and when these occurred, they were resolved through discussion.

##### 2.4.1.1 Positionality and Reflexivity

We recognise that qualitative data is co‐constructed by both participants and the interviewer (Finlay 2002). The interviewer, author MP is a clinical psychologist and early career researcher who has worked mostly with people with eating disorders. MP does not have IBD, however, has lived experience of chronic physical health and mental health conditions which influence her perspectives. Author IP was not involved in the co‐design groups but completed analysis alongside MP. IP is a researcher with expertise in body image and its application in a variety of health settings (including previous work with IBD populations). Both authors who conducted the analysis are white, heterosexual, cisgender women and able‐bodied. They are passionate about body image and inclusivity and championing improving body image and reducing weight and other appearance stigmas (while recognising their own privileges and biases). The authors met to discuss and reflect upon possible biases in their analysis where it occurred, allowing time in between meetings for reflection.

## 3 Results

Four key themes were developed to capture how IBD shapes body image as a dynamic, embodied, and context‐dependent experience: (1) Not Mainstream Body Image, (2) Functionality at the Core of Body Evaluation and Quality of Life, (3) Eating, Symptom Control, and Body Image, and (4) Unpredictability, Invisibility, and Social Appraisals. Across themes, body image was not experienced as a primarily appearance‐based construct, but rather as constructed through ongoing interactions between bodily function, symptom management, and social context. Theme summaries and illustrative quotes are provided in Table 2, while analysis and theme description are discussed below.

**Table 2:** Themes

Themes	Description	Illustrative Quotes
Not Mainstream Body Image	Body image was shaped primarily by illness‐related bodily changes and treatment effects, with appearance concerns reflecting disruptions to identity, control, and the visibility of illness rather than conventional appearance ideals alone.	“I had the ‘moon face’… it was literally the only reason why I ever grew facial hair was to be able to cover that.” “But one thing I've also struggled with, with the steroids was crazy hair loss and breakage of my hair. So, I used to have thick nice hair and now it's quite thin and wispy… the steroids then increased your hair growth in areas you didn't want it.” “It also affected my body image in terms of dissatisfaction, and it caused a negative view of the physical self of myself because, firstly, I get constant diarrhoea and it caused me to lose weight.” “… close to three months on steroids and I've put on 10 kilos and then I come off the steroids and I might lose like one or two… then have another flare and be put on steroids. Again, put on another 10 kilos and then only lose another couple before it happened again and I just progressively get bigger and bigger and I was just like what? What is the point? (of trying to lose weight) … my wardrobes goes from size 8 to size 18 and that's expensive.” “And as a female, it's especially hard because the society's pressures and pressures on yourself about weight gain. As a female, it's not as bad for a guy, I don't think. And yeah, it's just it's tough, especially if also you've had eating disorders too. It really impacts you.” “I don't even like my husband seeing me now without a T‐shirt on.”
Functionality at the Core of Body Evaluation and Quality of Life	Body image and wellbeing were fundamentally grounded in perceptions of bodily functioning, with experiences of reliability, loss, and adaptation shaping emotional responses, self‐evaluation, and broader life participation.	“My body “lets me down”… I feel like I'm deprived of being able to be normal like everybody else.” “And due to the initial surgeries I had so many pains in my back, I couldn't sleep properly. I couldn't sit. I can't stand.” “My fertility is down the **** creek now… the complications increase with my ulcerative colitis and so I unfortunately lost a baby last year.” “The sooner I now report I'm having a flare up, the lower the dose of the steroids and the shorter the stint. So, I'm learning that if I just go and tell them and get it over and done with and have the steroids, it's a shorter stint and I don't have as many side effects from it.” “Struggle to get out of bed, you know, not be able to meet up with people, just feel crap. Even doing the day‐to‐day.” “I can't go for a walk with you. We can't go down the beach and walk from (location) and back. Just it cannot happen. Whether I have an accident on the way or not, my brain is just not gonna be focusing on anything you say”. “I'm a mother. And I find, you know, I have IBD, and problems with my Crohn's. I struggle with that, with my children… I'm tired, but I need to get up. And like, the fatigue is a huge part of it… I feel that like, I can't keep up with a lot of other mums.” “I'm single and how much my body's changed. I stress about when I finally met someone I've lost a few relationships because of my health. One was I can't have kids now cause I've had the surgeries and its just too high risk and they wanted kids, I couldn't. At the moment I'm pretty much bedridden so I can't go out anywhere. I can't have anyone over, so I'm like a zombie.” “It's the mental side of things like, there'll be days that I feel good, like so this doesn't really both me, but then energy won't be there… I'm like, ohh this isn't working or I can't do anything. So it's like, what's the point?” “It affected my mental health as well. I felt so much like I need to be alone and even I'm alone I'm not feeling any better. It makes me feel less of a person. Like I lost my worth.” “I think the best thing I did was ostomy surgery. That's really, I have more certainty, and I can actually plan things now.” “Just do what you can manage, the things you need to do.” “Really the best thing for all of us is meeting people that have similar issues and realise that you're not the only one “So, you know, just accepting the new normal.”
Eating, Symptom Control, and Body Image	Eating behaviours functioned as a central mechanism for managing symptoms and control, creating a complex tension between adaptive dietary restriction, psychological burden, and the risk of disordered eating, all of which influenced body image.	“Every single day, what I eat, what I'm taking inside, I need to think that, OK that is causing problem. That'll have extra bleeding, extra pain. Yeah, so I think this is a psychological thing always in the brain.” “The mental load of deciding what I'm gonna eat is part of an issue that I have.” “I wasn't very much when I was like 11… I was just eating rice… And it's like, that kind of stuff when you're so young, it like, follows you through everything.” “I would basically starve myself before doing a long run, in fear that I wouldn't find a toilet, but I wanted to do the run. So now I can do 35 kms on an empty stomach for a day… you're not fuelling your body properly.” “We're going to pubs and stuff… I don't know if my stomach's great today, so I don't know if I should eat this, but I'm not gonna. It's too much like everyday.”
Unpredictability, Invisibility, and Social Appraisal	The unpredictable and invisible nature of IBD shaped body image through heightened self‐consciousness, social uncertainty, and anticipated judgement, with others' perceptions playing a key role in how individuals experienced and evaluated their bodies.	“That's the big thing that you don't look sick.” “… when there's five people waiting to use the toilets… I get a lot of abdominal pain because of it [waiting]… Do I just use the disabled toilet? But then what is somebody comes up and it's like, why is that?” “It's not a visible illness… everyday, um, fighting like, you know, really fighting with this condition and not anybody understands apart from your guys [study participants] and people that are living with this condition.” “We never know what's coming next.” “The unpredictability… nobody, nobody really, really gets it.” “It's uncertain, the uncertainty. Like, you don't what tomorrow's going to bring… Like I don't know if I'm going to feel up to it in an hour's time.” “Ohh, I wish I had what you have. You have something I'd love to lose that amount of weight” “It's unbelievable what comes out of their mouths, and they think that's acceptable… don't call me fat now when I have moon face.”

### 3.1 Not Mainstream Body Image

Body image concerns were grounded in illness‐specific bodily changes and treatment effects, rather than conventional appearance ideals alone. Participants described dissatisfaction with their appearance; however, this dissatisfaction was closely tied to disease‐related symptoms and treatment consequences such as steroid‐induced ‘moon face’, bloating, hair loss, growth of body hair, and surgical scarring. These changes disrupted how participants recognised and related to their bodies, contributing to a sense that their body was no longer stable or predictable.

Importantly, while broader sociocultural pressures (e.g., thin ideals) were present, these were often intensified through IBD‐specific bodily changes. For example, weight gain associated with steroid use was not only experienced as a deviation from appearance ideals, but also as a visible marker of illness and loss of control. Conversely, weight loss during disease flares was sometimes experienced ambivalently, reflecting tensions between illness severity and socially valued thinness. Participants also described engaging in body image‐related behaviours such as body checking and avoidance, often in response to illness‐related appearance changes. These behaviours were not solely driven by appearance dissatisfaction, but by attempts to manage distress associated with visible signs of illness and altered bodily identity.

Taken together, body image in this context was not adequately captured by traditional appearance‐based frameworks, but instead reflected an illness‐contingent evaluation of the body.

### 3.2 Functionality at the Core of Body Evaluation and Quality of Life

This theme reflects how body image and quality of life more broadly was fundamentally organised around perceptions of body functionality. Participants evaluated their bodies in terms of reliability, capability, and control, with disruptions to functionality directly shaping body‐related thoughts and emotions.

Participants commonly described feeling let down and disappointed by their bodies, particularly in relation to symptoms such as incontinence, pain, fatigue, and reproductive challenges. These experiences contributed to feelings of frustration, betrayal, and diminished trust in the body. In this way, body image was closely tied to functionality, with negative body image emerging when the body was perceived as unreliable or failing.

At the same time, some participants described increased bodily awareness as a form of adaptive engagement. Learning to detect early signs of flares and respond proactively contributed to a more nuanced relationship with the body, characterised by vigilance but also, at times, appreciation of what the body could still do. This highlights a tension between functional loss and functional attunement, both of which shaped participants' perceptions of their bodies.

Importantly, disruptions in functioning extended beyond the body itself into everyday life. Limitations in mobility, social participation, work, parenting, and relationships reinforced negative body evaluations, as participants compared themselves to others and perceived themselves as less capable or “normal”. Understandably, participants spoke of the impact of IBD and body functionality on mental health, including experiencing depression, anxiety, hopelessness, low self‐esteem, and difficulties managing uncertainty. Despite the significant challenges posed by IBD, some were able to accept the illness and their changed bodies. Contributing to this acceptance were strategies such as focusing on the things in one's control, connecting with others who have lived experience for support and validation, working on thinking in helpful balanced ways (rather than engaging in unhelpful thinking such as catastrophising), and striving for wellbeing.

### 3.3 Eating, Symptom Control, and Body Image

This theme captures how eating behaviours and dietary management were central to participants' experiences of body image and quality of life, reflecting a complex interplay between symptom control, psychological burden, and self‐perception. Participants described food as both necessary and threatening, requiring constant monitoring to avoid exacerbating symptoms. This resulted in a heightened cognitive and emotional load, where decisions about eating were experienced as stressful and effortful. Within this context, restrictive eating practices often initially emerged as adaptive strategies aimed at managing pain, urgency, or embarrassment. However, these practices also shaped subsequent body image. For some, restrictive or compensatory behaviours (e.g., fasting before exercise) led to concerns about not adequately nourishing the body, generating tension between maintaining control over symptoms and caring for the body's needs. In this way, body image was influenced not only by how the body looked or functioned, but also by how symptoms of IBD were managed.

Importantly, participants' accounts highlighted the need to distinguish between adaptive dietary management and potentially maladaptive eating behaviours. While some behaviours resembled disordered eating, they were often rooted in illness management rather than weight or shape concerns alone. Conversely, some participants spoke about having an eating disorder previously and dietary restriction (for the purpose of symptom control) as a slippery slope into disordered eating for weight and shape motivations. Thus, eating and nutrition were not peripheral concerns but central mechanisms through which body image and identity were constructed and negotiated in the context of IBD.

### 3.4 Unpredictability, Invisibility, and Social Appraisal

This theme reflects how the invisible and unpredictable nature of IBD shaped body image through social interactions, anticipated judgement, and difficulties in communicating illness experiences. The inability to anticipate symptoms or plan ahead contributed to a sense of instability and loss of control, which extended to participants' sense of self and their bodies. This unpredictability also affected engagement in social and relational contexts, including dating and intimacy, where concerns about disclosure, rejection, and bodily exposure were salient. Furthermore, participants described IBD as an “invisible illness,” which created a disconnect between their internal experiences and external appearance. This incongruence contributed to feelings of illegitimacy and anxiety about how others perceived them, particularly in situations where accommodations (e.g., using accessible toilets) might be questioned.

Body image was shaped not only by how participants saw themselves, but by how they believed others saw them. Experiences of stigma, misunderstanding, and inappropriate comments (e.g., about weight changes) reinforced negative body evaluations and heightened self‐consciousness. In this way, the social environment played a key role in constructing body image.

## 4 Discussion

The aim of this study was to qualitatively explore the impact of IBD on body image among individuals with heightened levels of body image concern. While conversation was initially focused on body image, it extended to quality of life and mental health and thus themes also encompass these areas and how these aspects are shaped by how the body is experienced and perceived for people with IBD. Thematic analysis yielded four main themes: Not Mainstream Body Image; Functionality at the Core of Body Evaluation and Quality of Life; Eating, Symptom Control, and Body Image; and Unpredictability, Invisibility, and Social Appraisals. Findings support previous qualitative work on IBD and body image in a Chinese sample (Ruan et al. 2020) and qualitative work in the area more broadly (Fourie et al. 2018). To the best of our knowledge, this study is the first qualitative exploration focused on body image and IBD in a Western country. Our findings extend current knowledge in several ways.

Our themes capture the unique ways body image is impacted and shaped by IBD. People with IBD experience body dissatisfaction that is directly related to IBD symptoms and treatments, such as weight changes from flares and medications, “moon face” from taking steroid medication, stomach bloating, hair loss and changes, and scars from surgery. This finding is consistent with previous qualitative research themes related to having a flawed body (Erdmann et al. 2024; Fourie et al. 2018; Ruan et al. 2020). In addition, participants in our study spoke of ‘feeling fat’, fearing weight‐based judgement from others, and body image behaviours such as body checking and body avoidance, all of which are common features in eating disorders (Fairburn 2008; Waller et al. 2007). This is an important finding that highlights the overlap between IBD and eating disorders, with disordered eating estimated to be prevalent in between 13% to 29% of IBD patients (Satherley et al. 2016; Wabich et al. 2020; Wardle et al. 2018). To the best of our knowledge, while participants in our study had high levels of body dissatisfaction, there are currently no qualitative explorations of IBD and eating disorders. Given the estimated high prevalence of eating disorders in the IBD population, this is an important avenue for future research. Indeed, the relationship between IBD and eating disorders has been described as “bi‐directional and complex” (Staller et al. 2023) and warrants further attention.

Body functionality is at front of mind for people with IBD and actively shaped body image and life experiences. Participants often spoke of feeling disappointed and let down by their bodies in addition to specific challenges such as anxiety about incontinence. Functionality tension and body disappointment has also been described in previous qualitative research in IBD (Erdmann et al. 2024; Fourie et al. 2018; Ruan et al. 2020). Body functionality appreciation plays an important role in positive body image, health and wellbeing (Alleva and Tylka 2021), as demonstrated by body functionality appreciation writing interventions that significantly improved positive body image with small to medium effect sizes at follow‐up in general populations (Alleva et al. 2018; Alleva et al. 2018; Alleva et al. 2015). Thus far, there are no investigations of how body functionality appreciation relates to people with IBD. Promisingly, body functionality is of relevance, and intervention has been effective, in other areas of health such as cancer (Brkic et al. 2024; Ettridge et al. 2022). Targeting body functionality, whilst also recognising the tension people with IBD feel in relation to their body's function, is a recommended future pursuit to help people with IBD increase their positive body image.

Food choices, eating behaviours, and their use in controlling symptoms were prominent in our sample and actively shaped body image and quality of life. Participants highlighted the stress and mental load attributable to deciding what to eat and how this then impacts upon their IBD symptoms, enjoyment of food, social life and eating out, in addition to discussions of disordered eating. This theme extends upon the overarching “Living with Restriction” theme identified by Fourie et al. (2018), offering greater insight into the mental toll dietary restriction creates. Nutrition interventions and dietary adaptations are common as part of holistic IBD treatment, however a systematic review and meta‐analysis of prospective controlled trials demonstrated mixed effectiveness (Limketkai et al. 2023). For CD, there was some evidence (of low certainty) demonstrating a relationship between the use of partial enteral nutrition and both the induction of remission and reduced risk of relapse while for UC, there was no clear evidence of benefit of any dietary intervention (Limketkai et al. 2023). Thus, it is understandable that knowing what to eat and the use of dietary intervention can create considerable stress. For instance, Day et al. (2022) found food‐related quality of life was worse for patients with IBD who had restrictive eating and clinically active disease. Furthermore, a recent review of six qualitative studies of IBD and dietary experiences demonstrated that it is common for people with IBD to completely change their diet in an attempt to manage symptoms, despite there not being consistent guidance on how best to achieve this (Xiong et al. 2024). This highlights the emotional and mental toll faced by people with IBD regarding their dietary changes (Xiong et al. 2024) and the need for better guidance and support to manage nutrition and the psychological impacts of modifying diet. Clinicians should consult the recently published European Crohn's and Colitis Organisation consensus on dietary management of inflammatory bowel disease (Svolos et al. 2025) for a summary of the evidence to date and recommendations for dietary intervention.

The current study is not without limitations. First, participants were all recruited from the same IBD service in South Australia and thus may not be representative of all patients with IBD. Second, participants all had a BIS score of 10 or more and thus represented a group of particularly body‐dissatisfied people with IBD. Third, our sample size precluded an analysis specific to gender. We noted that participants discussed similar content regardless of gender, however we cannot make firm conclusions about this given only two male participants were involved. It will be important for future research to continue strengthening and expanding upon the present study with diverse samples.

Notwithstanding these limitations, our findings expand upon the limited literature qualitatively exploring the impact of IBD and body image (Ruan et al. 2020) in addition to qualitative explorations of the impact of IBD more broadly (Erdmann et al. 2024; Fourie et al. 2018). This was the first study, to our knowledge, to exclusively focus on the impact of IBD on body image in a Western country and the first to identify content related to disordered eating and eating disorders. As such, it is a first step in developing mental health protocols and treatments tailored to people with IBD and body image concerns. It will be important for future research to further explore the relationship between IBD and eating disorders using a qualitative design to better understand this common comorbidity. Interventions that target body image for people with IBD is an important endeavour, particularly those that incorporate body functionality. We also encourage future work on nutrition and dietary management of IBD to incorporate food‐related quality of life and evaluate the psychological impacts of dietary intervention.

## Ethics Statement

This study received ethics approval from the Southern Adelaide Clinical Human Research Ethics Committee (project number 2023/HRE00016).

## Consent

All participants provided informed consent.

## Conflicts of Interest

R.M. and R.P. are employed by SALHN and M.P. was employed by SALHN at the time the grant was announced. I.P. has no conflicts of interest to declare.

## Data Availability

The participants of this study did not give written consent for their data to be shared publicly, so due to the sensitive nature of the research supporting data is not available.
